# The variant Polycomb Repressor Complex 1 component PCGF1 interacts with a pluripotency sub-network that includes DPPA4, a regulator of embryogenesis

**DOI:** 10.1038/srep18388

**Published:** 2015-12-21

**Authors:** Giorgio Oliviero, Nayla Munawar, Ariane Watson, Gundula Streubel, Gwendolyn Manning, Vivian Bardwell, Adrian P. Bracken, Gerard Cagney

**Affiliations:** 1 School of Biomolecular and Biomedical Science, University College Dublin, Belfield, Dublin 4, IRELAND; 2 Department of Genetics, Trinity College Dublin, Dublin 2, IRELAND; 3 Developmental Biology Center, Masonic Cancer Center, and Department of Genetics, Cell Biology, and Development, University of Minnesota, Minneapolis, MN 55455 USA

## Abstract

*PCGF1* encodes one of six human Polycomb RING finger homologs that are linked to transcriptional repression and developmental gene regulation. Individual PCGF proteins define discrete Polycomb Repressor Complex 1 (PRC1) multi-protein complexes with diverse subunit composition whose functions are incompletely understood. PCGF1 is a component of a variant PRC1 complex that also contains the BCL6 co-repressor BCOR and the histone demethylase KDM2B. To further investigate the role of PCGF1, we mapped the physical interactions of the protein under endogenous conditions in a cell model of neuronal differentiation. Using stringent statistical cut-offs, 83 highly enriched interacting proteins were identified, including all previously reported members of the variant PRC1 complex containing PCGF1, as well as proteins linked to diverse cellular pathways such as chromatin and cell cycle regulation. Notably, a sub-network of proteins associated with the establishment and maintenance of pluripotency (NANOG, OCT4, PATZ1, and the developmental regulator DPPA4) were found to independently interact with PCGF1 in a subsequent round of physical interaction mapping experiments. Furthermore, knockdown of PCGF1 results in reduced expression of DPPA4 and other subunits of the variant PRC1 complex at both mRNA and protein levels. Thus, PCGF1 represents a physical and functional link between Polycomb function and pluripotency.

The regulation of gene expression through epigenetic mechanisms operates at several levels. These include modification of DNA itself, modification of the histone proteins in contact with DNA, as well as higher order regulation involving ‘remodelling’ and three-dimensional rearrangement of chromosomes to increase or decrease accessibility to the DNA by transcription factors^1^. Many of these changes are mediated by large heteromeric protein complexes possessing multiple activities that ensure that specific epigenetic changes occur at particular genes at the correct time.

One family of such complexes are known as Polycomb Repressive Complexes (PRC)^2^,^3^. The genes encoding components of these complexes were originally isolated in genetic screens of *Drosophila*, where mutants display a variety of developmental phenotypes, suggesting a critical role for Polycomb genes in the regulating of genes involved in cell fate determination and differentiation^4^,^5^. Later biochemical work established that Polycomb function was mediated by two classes of multi-protein enzymatic complexes (PRC1 and PRC2) whose central catalytic functions include histone post-translational modification activities (i.e. ubiquitin ligase and methyltransferase activity respectively). The catalytic core of PRC1 in humans is a heterodimer comprising either of two related E3 ubiquitin ligases, RING1 (RING1A) or RNF2 (RING1B) and one of six PCGF orthologs^6^,^7^,^8^,^9^,^10^.

Additional core components include a chromodomain-containing protein (CBX2, CBX4, CBX6, CBX7 or CBX8) and a Polyhomeotic protein (PHC1, PHC2, or PHC3)^6^,^10^,^11^,^12^. To date only PCGF2 and PCGF4 among the six PCGF orthologs have been found in this canonical form of PRC1^10^,^13^. An alternative complex, termed ‘non-canonical’ or ‘variant’ PRC1 (vPRC1), was isolated by Gearhart and coworkers and contains PCGF1, RING1, RNF2, RYBP, BCOR, SKP1, and KDM2B^14^. Notably, this variant complex houses proteins capable of both H2BK119 ubiquitination and H3K36 demethylation, suggesting a multifunctional role for vPRC1 linked to gene silencing^14^.

The mechanism by which PCGF1/PRC1 is recruited to target genes is not fully understood, but one component, KDM2B (also called FBXL10) houses a Zf-CxxC domain that has affinity for CpG-rich DNA^15^,^16^. Furthermore, ChIP-seq experiments with KDM2B found that it localizes to CpG islands and is often co-located with RNF2^15^,^16^,^17^,^18^. A subset of all PRC1 targets are reported to be occupied by PCGF1/PRC1^10^ and recently, Blackledge and coworkers showed that recruitment of PCGF1/PRC1 results in H2A K119 ubiquitylation which is required for subsequent recruitment of PRC2 and deposition of H3K27me3^19^,^20^ They also showed that the KDM2B-mediated targeting activity was required for normal mouse development^19^. Transcription factors can also contribute to the recruitment of vPRC1. In mature B cells, BCL6 was shown to play a role in recruiting the PCGF1/BCOR/PRC1 complex^21^.

The combination of affinity purification and high resolution/high mass accuracy mass spectrometry has significantly increased the power of protein interaction mapping experiments in recent years. Exogenously expressed affinity-tagged proteins can be captured using reagents that recognize tags such as FLAG, HA, and poly-histidine. Alternatively, immunoprecipitation using antibodies specific to the target protein can be used, with the advantage that the endogenous protein is sampled in its native molecular environment. Affinity tagging approaches were used to identify interactors of PCGF1 and the five other PCGF homologs^10^,^14^,^15^. These experiments yielded important insights into the composition of PCGF-containing complexes (including the role of the RYBP or YAF2 subunits in defining variant complexes that do not contain CBX SCM-like and PHC proteins) and linked alternative PRC1-related complexes to distinct epigenetic functions. The authors employed exogenous expression of the target protein that may not completely reflect native conditions. Here we used α-PCGF1 antibody that is efficient as an immunoprecipitation reagent to purify PCGF1 from NT2 embryonic carcinoma cells, yielding all known components of the variant PCGF1-PRC1 complex, as well as many new potential interactors. Notably, the approach identified a sub-network of pluripotency-associated proteins that interact with PCGF1, including DPPA4, which we found to be under PCGF1 regulatory control.

## Results

### A physical interaction screen for PCGF1 under endogenous conditions

**Figure 1:**
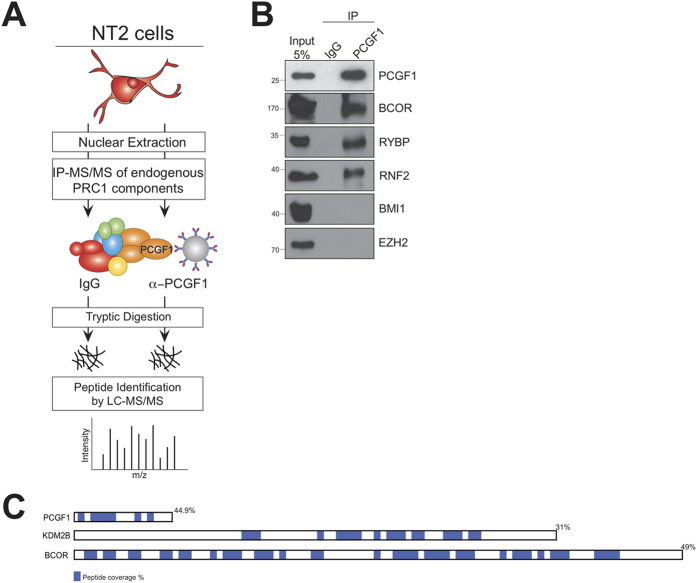
A physical interaction screen for PCGF1 under endogenous conditions. (**A**) PCGF1 and interacting proteins were immunoprecipitated from NT2 cell nuclear lysate and analysed by label-free mass spectrometry. (**B**) Input lysate and samples immunoprecipitated using control (IgG) and specific (PCGF1) antibody were analysed by western blot using antibodies to the ‘bait’ protein PCGF1, two previously described members of the vPRC1 complex (BCOR, RYBP), and representative members of the canonical PRC1 (PCGF4/BMI1) and PRC2 (EZH2) complexes. (**C**) Amino acid sequence coverage by tandem mass spectrometry is indicated (blue and percentage) for vPRC1 components PCGF1, KDM2B, and BCOR.

We optimized the experimental conditions needed to purify PCGF1-interacting proteins from undifferentiated NT2 cells (Fig. 1A). NT2 is a human teratocarcinoma cell line capable of differentiation to neuron and glia phenotype upon treatment with all-*trans* retinoic acid^17^. These cells express PCGF1 (Fig. 1B) and are a good model of Polycomb regulation of neuronal differentiation genes^20^. Briefly, nuclear lysates were prepared from undifferentiated NT2 cells, PCGF1 and its interacting partners were immunoprecipitated, and digested using trypsin *in situ* on agarose beads to yield soluble peptides. The peptides were desalted, adsorbed onto C18 zip tips, eluted in high acetonitrile, and separated online by nano-chromatography interfaced with a Q Exactive mass spectrometer (Supplementary Table S1). α-PCGF1 but not IgG immunopurified lysates contained PCGF1 and the variant PCGF1/PRC1 complex components BCOR, RNF2, and RYBP, indicating efficient immunoprecipitation (Fig. 1B). Notably, the canonical PRC1 component PCGF4 (BMI1), and the PRC2 methyltransferase EZH2 were not detected in the purified lysates (Fig. 1B). High peptide coverage of known members of the variant PRC1 complex showed that the mass spectrometry experiments were sufficiently sensitive to reliably detect PCGF1 and its interaction partners (Fig. 1C).

### PCGF1 co-purifies with members of the variant PCGF1-PRC1 complex, as well as additional proteins linked to diverse cellular processes

**Figure 2:**
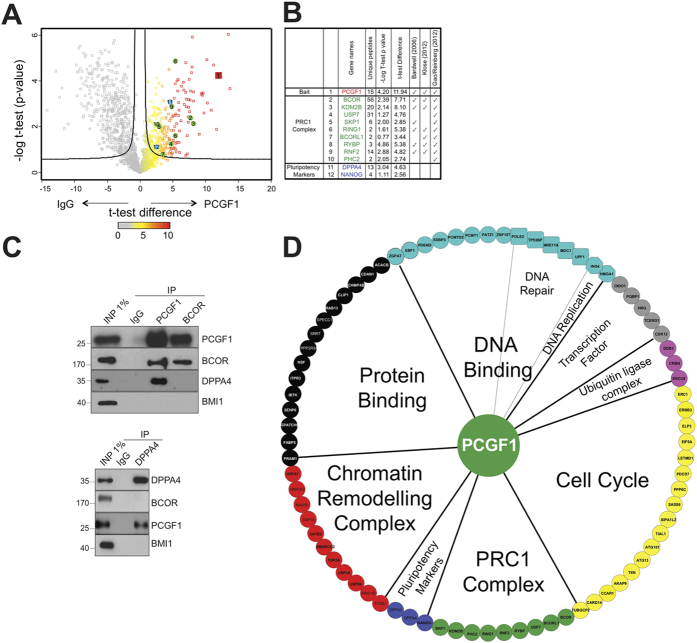
PCGF1 co-purifies with members of the variant PCGF1-PRC1 complex, as well as additional proteins linked to diverse cellular processes. (**A**) Proteins enriched by PCGF1 immunoprecipitation are shown in Volcano diagrams that plot t-test difference versus P-value for enrichment in IgG or PCGF1 immunoprecipitation experiments. Bait (PCGF1) is shown in red, previously described members of vPRC1 in green, and pluripotency factors in blue. (**B**) Mass spectrometry protein coverage and previous reports in the literature for PCGF1-interacting proteins. (**C**) Co-immunoprecipitation experiments whereby PCGF1, BCOR and DPPA4 are pulled-down and probed with antibodies to PCGF1, BCOR, DPPA4 and BMI1. D. Network visualization of the PCGF1 interactome. Functional categories were defined using the Cytoscape plugin BiNGO^22^.

Protein abundance was determined by label-free mass spectrometry and used to compare samples immunoprecipitated using α-PCGF1 from samples immunoprecipitated in parallel experiments using mouse agarose beads (IgG) (Supplementary Table S2) as a negative control. Volcano plots project data describing the enrichment of proteins in an immunoprecipitated sample, and the statistical significance of that enrichment (t-test P-value), onto two dimensions (Fig. 2A,B). To confirm these results, we carried out co-immunoprecipitation experiments on PCGF1 precipitates using antibodies to PCGF1, BCOR, and a newly detected interactor, DPPA4 (Fig. 2C). None of the precipitated proteins were found to interact with the canonical PRC1 component PCGF4, demonstrating the specificity of the interaction. These MS data confirmed strong recovery of PCGF1 itself, all previously reported members of the PCGF1/PRC1 complex and 74 additional proteins (Supplementary Table S1).

The set of PCGF1 interacting proteins was analysed for enrichment in annotated functional properties using the BiNGO Gene Ontology network tool^22^, and the functional categories are summarized in a network representation of the interacting proteins centred on PCGF1/PRC1 (Fig. 2D). These potential interactors include members of other epigenetic regulatory assemblies such as SWI/SNF Chromatin Remodelling Complex (SMARCC2, ARID1B), normally considered to interact antagonistically with Polycomb proteins^3^,^23^, cell cycle related proteins (SASS6, LETMD1), DNA replication and repair proteins (MRE11A, DDB1), as well as proteins linked generally to protein and RNA binding. Interestingly, the well-known pluripotency factor NANOG was found to interact with PCGF1 in our experiments, as well as other proteins linked to pluripotency such as DPPA4 and PATZ1^24^,^25^. Although the functional interdependency of Polycomb genes and regulators of pluripotency is well known^26^, and it was recently shown that PCGF1 can regulate expression of OCT4 through binding of its promoter^27^, we are unaware of earlier reports of a physical association between a PCGF protein and NANOG, DPPA4 or PATZ1. In summary, our experiments confirmed that PCGF1 interacts with the variant PRC1 complex component BCOR. In contrast, DPPA4 interacted with PCGF1, but not BCOR, suggesting that it is not a member of either canonical or variant forms of the PRC1 complex.

### Analysis of molecular mass and relative stoichiometry of PCGF1-containing complexes

**Figure 3:**
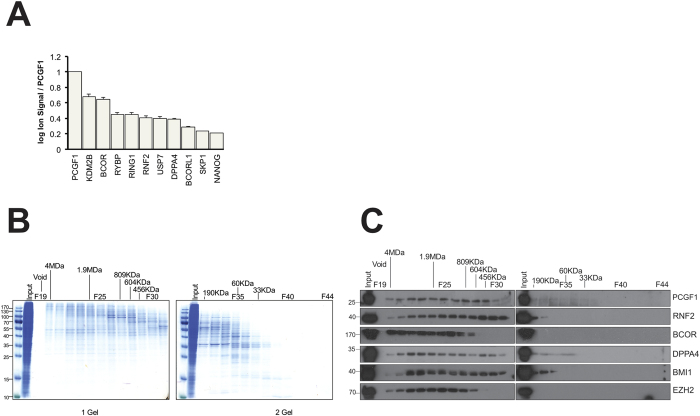
Analysis of molecular mass and relative stoichiometry of PCGF1-containing complexes. (**A**) Stoichiometry of PCGF1-interacting proteins was determined using extracted ion signal from MS experiments and plotted relative to the signal for PCGF1. (**B**) Size exclusion fractions were further separated by SDS-PAGE and stained with Coomassie blue. (**C**) Western blot analysis of size exclusion fractions for PCGF1-interacting proteins and controls.

We next focused on describing the physical PCGF1-containing complex(es). We estimated the relative subunit stoichiometry of PCGF1 to its interaction partners using mass spectrometry ion signal, and we used size exclusion chromatography to attempt to resolve complexes of differing size. Ion signal intensity can serve as a semi-quantitative measure of relative protein content by summing the intensities recorded for peptides unique to parent proteins, adjusting for protein size and the presence of computationally predicted ionizable peptides, and normalizing the data to the targeted protein^28^ (Fig. 3A). This analysis suggests that the variant PCGF1/PRC1 complex is the main location for PCGF1 in undifferentiated NT2 cells since it purifies in high ratios with KDM2B, BCOR, and other PRC1 components. Lower amounts of co-purifying DPPA4, NANOG, and SKP1 were observed, suggesting sub-stoichiometric but significant interactions. Pairs of proteins that interact at low stoichiometry can indicate that an interaction is transient or of low affinity (e.g. enzymes and substrates), or that the two proteins are present together in a complex of low abundance. In an attempt to investigate these alternatives, total protein lysate from NT2 cells was separated by size exclusion chromatography (Fig. 3B) and the fractions probed using antibodies for PCGF1, BCOR, RING1, DPPA4, PCGF4, CBX8, and EZH2. All these proteins were found to be present in high mass complexes with slight differences in distribution profile (Fig. 3C).

In our experiment, DPPA4 showed an elution pattern distinct from that of PCGF1. DPPA4 appeared to be present in a high mass complex of ~2 MDa, a smaller complex of ~300 KDa, and in a low mass form (~50–100 KDa). The high mass complex containing DPPA4 may be similar or identical to variant PCGF1/PRC1, since it has a similar elution profile to BCOR, and is similar to the PCGF1-containing vPRC1 described by others, for example those observed when affinity-tagged PCGF proteins were stably expressed in 293TREx cells by Gao and coworkers^10^. In other words, in this scenario DPPA4 would represent a new member of the vPRC1 complex. However, this seems not to be the case, since DPPA4 and BCOR did not co-precipitate with each other, raising the possibility that DPPA4 co-purifies with PCGF1 in a high mass complex distinct from variant PRC1. The total mass of a complex containing single copies of PCGF1, BCOR, KDM2B, RNF2, and RYBP (30, 192, 152, 38, 25 KDa respectively) would be approximately 440 KDa. The size exclusion experiments do not formally prove that the high mass complexes that contain PCFG1 and DPPA4 are in fact overlapping. The stoichiometry data (Fig. 3A) argue that <50% molar proportion of PCGF1 is associated with DPPA4. Furthermore, antibody to DPPA4 weakly stained eluate from Fraction 35, where protein markers of molecular mass 60 KDa eluted. Therefore, DPPA4 may participate in multiple assemblies, some very large, but also a small complex that could represent a dimeric form of DPPA4 (2 × 33KDa), or potentially a heterodimer with PCGF1 (30 + 33 KDa). Additional biophysical experiments will be needed to investigate further.

### A pluripotency-associated sub-network linked to PCGF1

**Figure 4:**
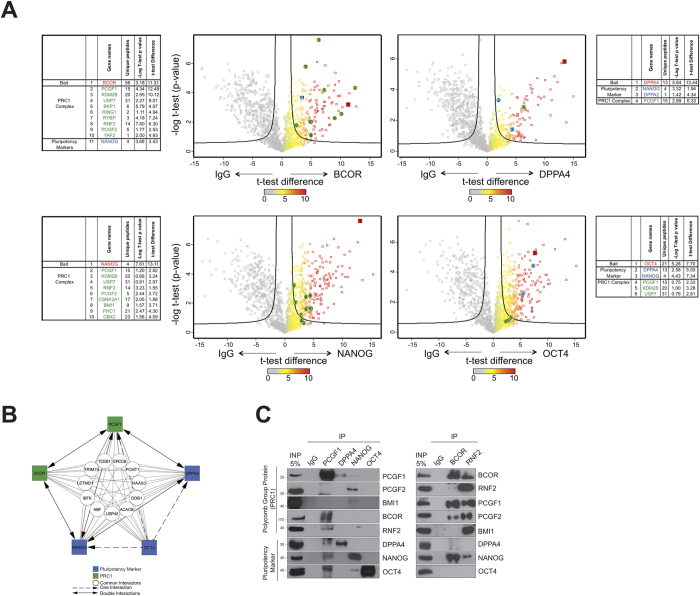
A pluripotency-associated sub-network linked to PCGF1. (**A**) Volcano diagrams for immunoprecipitations of BCOR, DPPA4, NANOG and OCT4. Baits are shown in red, previously described members of vPRC1 in green, and pluripotency factors in blue. (**B**) Network diagram of reciprocal and non-reciprocal physical interactions detected in affinity purification mass spectrometry experiments with the tail of the arrow on the immunoprecipitated protein. (**C**) Co-immunoprecipitation of PCGF1, DPPA4, NANOG, OCT4, BCOR, and RNF2 with western blot staining for PCGF1, PCGF2, BMI1, BCOR, RNF2, DPPA4, NANOG and OCT4.

Since the NT2 cell model displays a partial pluripotency phenotype (for example the expression of high levels of *NANOG* and *OCT4* and the repression of differentiation genes such as *HOXA9*), we reasoned that the sub-network of proteins linked to maintenance of pluripotency (NANOG, DPPA4, PATZ1) may share interaction partners with PCGF1. Although we did not detect it in our initial PCGF1 physical interaction screen, the pluripotency gene *OCT4* has also been functionally linked to Polycomb function^27^,^29^,^30^. Therefore we carried out four physical interaction mapping experiments identical to the PCGF1 immunoprecipitation/MS experiment but this time using antibodies to NANOG, OCT4, DPPA4, and BCOR (a component of the variant PCGF1/PRC1 complex). In all four immunoprecipitation/MS experiments, PCGF1 was found to be present using high stringency volcano plot analysis (Fig. 4A,B; Supplementary Table S3). Although neither PCGF1, nor NANOG, nor DPPA4, co-purified with OCT4 when those proteins were immunoprecipitated, the opposite was not the case – PCGF1, DPPA4 and NANOG were all detected in OCT4 immunoprecipitates by mass spectrometry, although the interaction of OCT4 with DPPA4 was below the FDR cut-off.

PCGF1, BCOR, DPPA4 and NANOG showed distinct patterns of association with the variant PCGF1/PRC1 complex (Fig. 4A). As expected, PCGF1 and BCOR shared an association with multiple members of the complex (KDM2B, RYBP, RNF2, USP7, SKP1). NANOG also co-purified with variant PRC1 members KDM2B, RNF2, USP7, as well as proteins linked to canonical PRC1 complexes such as PCGF2, PCGF4, CSNK2A1, PHC1 and PHC2. This suggests that NANOG physically associates with multiple distinct Polycomb complexes in undifferentiated NT2 cells. Notably, apart from PCGF1 itself, known subunits of the PCGF1/PRC1 complex were absent from DPPA4 and OCT4 immunoprecipitation experiments. The interactions of PCGF1 with BCOR, RNF2, DPPA4, NANOG and OCT4 were confirmed by co-immunoprecipitation analysis using α-PCGF1 (Fig. 4C). The signal from this co-immunoprecipitation experiment was noticeably weak (and in the case of DPPA4 associated with a doublet band, possibly due to a gel effect). For this reason, we confirmed the interaction between PCGF1 and DPPA4 by exogenously expressing a FLAG-tagged PCGF1 in NT2 cells and western blotting for using antibody to DPPA4 (Supplementary Fig. S1).

### Investigation of the PCGF1 pluripotency sub-network in a neuronal differentiation model

**Figure 5:**
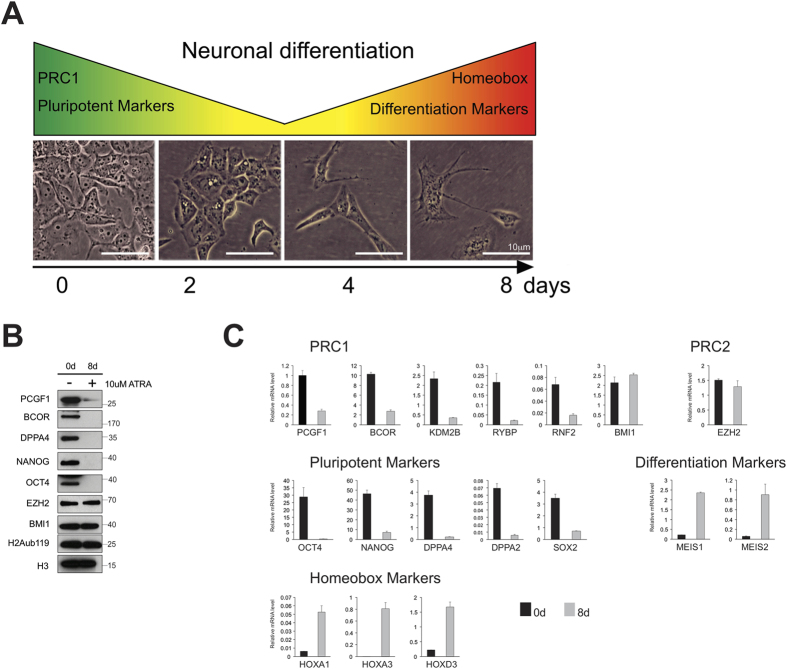
Investigation of the PCGF1 pluripotency sub-network in a neuronal differentiation model. (**A**) The NT2 cell differentiation model. (**B**) Protein expression of PCGF1-interacting and control proteins before and after addition of retinoic acid. (**C**) mRNA expression of genes before (0d) and after (8d) addition of retinoic acid.

In order to investigate potential functional links between PCGF1 and the pluripotency factors DPPA4, NANOG, and OCT4, we used the NT2 model of cellular differentiation (Fig. 5A). We confirmed that the model displays a differentiation phenotype by showing down-regulation of the pluripotency factor OCT4 (POU5F1), and up-regulation of the neuronal Polycomb target gene differentiation markers such as *MEIS1*, *MEIS2* and *HOX* genes following addition of retinoic acid (Fig. 5C).

Moreover, the differentiated cells show an elongated morphology distinct from that of undifferentiated cells (Fig. 5A). In order to confirm that DPPA4, NANOG, OCT4 and BCOR were differentially expressed, nuclear lysates were analyzed using western blotting with relevant antibodies (Supplementary Table S2). These experiments confirmed that DPPA4, OCT4 and NANOG were all strongly or completely down-regulated following differentiation, at the level of both protein (Fig. 5B) and mRNA (Fig. 5C). In contrast, neither the canonical PRC1 subunit PCGF4, nor the PRC2 subunit EZH2, were down-regulated, suggesting that PCGF1 plays a specific role in NT2 cell differentiation that is distinct from the roles of other PCGF homologs or that of the PRC2 complex. Global levels of the PRC1 substrate histone lysine 119 ubiquitination (H2AK119Ub) were unchanged (Fig. 5B).

### PCGF1 expression correlates with DPPA4 expression in NT2 cells

**Figure 6:**
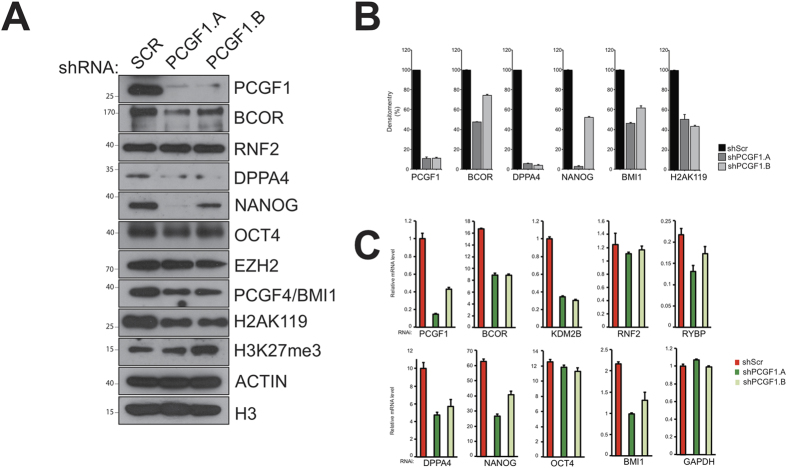
PCGF1 regulates DPPA4 expression in NT2 cells. (**A**) Protein expression of PCGF1-interacting and control proteins following disruption of PCGF1 expression using shRNA. (**B**) Quantification of the protein levels in panel A. (**C**) mRNA expression for genes encoding PCGF1-interacting and control proteins following disruption of *PCGF1* expression using shRNA.

Since PCGF1 shows similar expression patterns to DPPA4 and NANOG, and physically associates with them, we asked whether PCGF1 might serve as a regulator of DPPA4 or NANOG in NT2 cells. Knock-down of *PCGF1* resulted in reduced levels of NANOG and DPPA4 (but not OCT4) protein, raising the possibility of involvement of PCGF1 in the regulation of these pluripotency factors (Fig. 6A,B).

Reduced *PCGF1* also led to reduced protein levels of the variant PRC1 subunit BCOR, but not of the RNF2 subunit. BCOR that is affinity purified from HeLa cells primarily co-purifies with a complex very similar to the variant PCGF1/PRC1 complex^14^. In contrast, RNF2 has been purified in the context of several different multi-protein complexes^31^, perhaps explaining why these two proteins show different behaviour following knockdown of PCGF1. Interestingly, knockdown of PCGF1 also resulted in reduced levels of PCGF4 but not of PRC2 subunit EZH2, suggesting a level of auto-regulatory activity among PCGF genes. Furthermore, even though levels of RNF2 were not reduced following knockdown of PCGF1, we noted a small but detectable decrease in the levels of global H2AK119ub. This could arise from reduced levels of either variant PCGF1/PRC1 or of canonical PCGF4/PRC1, both of which mediate H2A K119 ubiquitination.

The reduced NANOG and DPPA4 that we observed could originate from: a) reduced mRNA expression because PCGF1 is a direct or indirect regulator of the downstream genes, or b) increased degradation of NANOG and DPPA4 protein in the absence of a (direct or indirect) stabilizing interaction with PCGF1. In order to investigate these possibilities, we used qPCR to measure the expression of mRNA following knockdown of *PCGF1* (Fig. 6C) and found gene expression levels to broadly correlate with protein levels. mRNA of the variant PCGF1/PRC1 subunits *BCOR*, *KDM2B*, and *RYBP* were significantly down-regulated, but *RNF2* mRNA was only modestly down-regulated. The pluripotency factors NANOG and DPPA4 were down-regulated at the protein level, as was PCGF4 to a lesser degree. For NANOG and DPPA4 the reduction in protein expression appeared to be more profound than the reduction in mRNA levels (one of the shRNAs used for PCGF1 was incompletely effective). This raises the possibility that PCGF1 is involved in the regulation of *NANOG* and *DPPA4* at the level of transcription. However, ChIP experiments designed to investigate this did not perform in our hands using this antibody.

## Discussion

In order to map the physical interactome of PCGF1 in a context as close as possible to its native environment, we used an endogenous immunoprecipitation approach in a cell line with neural progenitor cell properties. We detected an interaction between PCGF1 and DPPA4 that seems to be independent of other members of the vPRC1 complex. Both DPPA4 and a related family member, DPPA2 (35% amino acid sequence identity), encode SAP (Scaffold attachment factor – Acinus – Protein inhibitor of activated STATs) domains, are close together on chromosome 16, and are expressed in pluripotent cells and developing germ lines^25^. One or both of these genes have been detected repeatedly in screens for pluripotent activity^32^,^33^,^34^,^35^. To our knowledge, DPPA4 has not previously been described in connection with Polycomb biology, apart from a potential physical interaction between the DPPA4 and the Polycomb protein L3MBTL2 listed in the supplementary material of a large-scale screen of human protein-protein interactions^36^. Although we did not detect this protein among PCGF1 interactors, L3MBTL2 was recently found to be a component of a PRC1-related complex isolated from stem cells^37^,^38^. Taken together, our mass spectrometry and co-immunoprecipitation experiments argue that a modest fraction (perhaps 5–10% based on stoichiometry analysis, Fig. 3A) of PCGF1 is complexed with DPPA4 and NANOG in cultured NT2 cells, but that conversely, the majority of DPPA4 and NANOG is present independently of PCGF1. This is consistent with a regulatory relationship between PCGF1 and the pluripotency factors, whereby the interaction is either transient, or is present in a subset of loci occupied by PCGF1-PRC1.

Knockdown of *DPPA4* using shRNA was previously found to decrease expression of pluripotency markers like *OCT4*, *NANOG* and *REX-1* in differentiating ES cells, and this result (along with a finding that DPPA4 binds the promoter regions of *OCT4* and *NANOG*) was interpreted as indicating a role for DPPA4 in ES cell renewal and inhibition of differentiation markers^33^. However, later work using ES cell lines and homozygous mutant mice found no change in expression of pluripotency markers arising from loss of *DPPA4*, nor evidence for an essential role in self-renewal or pluripotency^34^. These authors did find that *DPPA4* null mice died late embryonically/perinatally and displayed defects in the skeletal development, which suggests that DPPA4 plays an important developmental role. However as the allele was not conditional so they were unable to test for a maternal effects which could manifest at a very early developmental stages. Interestingly, *DPPA4* was also recently found in a screen for oncogenic foci-inducing genes^35^. The authors confirmed the finding in transformation assays in NIH3T3 cells and immortalized fibroblasts, and in an immunodeficient mouse tumor model. The mechanism of enhanced proliferation appeared to involve regulation of the G1/S cell cycle checkpoint, since overexpression of *DPPA4* led to up-regulation of transcripts for genes such as *CCNB1*, *CCND1*, *E2F1*, and *MTBP*. Variant PRC1 itself has been implicated in the regulation of cellular proliferation. The variant PRC1 protein KDM2B was found to influence the regulation of proliferation and senescence through regulation of the *INK4A* and *INK4B* loci^39^,^40^,^41^. Although the details of how the function of variant PRC1 or DPPA4 influence cellular proliferation pathways are unclear, both hESCs and iPSCs have tumor promotion properties^42^, while the importance of cancer stem cells in both solid and liquid tumors is now well recognized^43^.

A notable outcome of our work is the finding that disruption of PCGF1 expression leads to decreased expression of: a) some, but not all, members of the variant PRC1 complex; b) at least one other PCGF ortholog (and component of a canonical PRC1 complex); and c) pluripotency factors including DPPA4 and NANOG. The presence of PCGF1 is associated with active expression of NANOG and DPPA4. It is unclear if our data indicates that PCGF1 directly stimulates transcription of *NANOG* and *DPPA4*, or acts indirectly, for example by repressing expression of a negative regulator of *NANOG* and *DPPA4*. The observations raise the possibility of the presence of a network of interrelated feedback loops that serve to regulate gene expression among the network. In this context, the recent finding that over- and under-expression of PCGF1 correlates with expression of pluripotency factors NANOG, OCT4 and SOX2 in P19 embryonal carcinoma cells is of interest^27^. The nature and dynamics of such auto-regulatory loops are currently unknown, however evidence from computational modelling approaches suggest an important role for feedback loops in Polycomb biology^44^. Although Polycomb complexes are generally associated with repression of gene expression, the presence of multiple interconnected regulatory feedback loops may explain the counter-intuitive observation that decreased PCGF1 leads to reduced expression of its ortholog *PCGF4* at the level of mRNA expression. For example, PCGF2 as been shown to repress *PCGF4* in human fibroblast cells^45^.

If such a mechanism operates in NT2 cells, then decreased PCGF2 expression arising from PCGF1/PRC1-mediated repression could plausibly lead to increased *PCGF4* expression. By corollary, disruption of *PCGF1* activity in our shRNA experiment could lead to increased *PCGF2* and therefore reduced *PCGF4* expression. Further genetic and biochemical studies will hopefully shed light on the direction and reciprocity of Polycomb-Polycomb and Polycomb-Target regulatory mechanisms within pluripotency networks, the mechanisms by which they operate (i.e. direct or indirect), and the nature of the PRC1-related complexes involved.

## Experimental Procedures

### Cell culture

HEK293T cells (CRL-1573, ATCC) were cultured in 92 mm Nunclon tissue culture dishes (Fisher Scientific) in Dulbecco’s Modified Eagle Medium (DMEM) supplemented with 10% (v/v) Fetal Bovine Serum (Hyclone), 100 U/ml penicillin and 100 U/ml streptomycin (Gibco). Cells were passaged by trypsinizing with 0.25% Trypsin-EDTA (Invitrogen) and plated at a ratio of 1:10. NT2/D1/D1 cells (ATCC, CRL-1973) were cultured in Dulbecco’s Modified Eagle Medium (DMEM) supplemented with 10% (v/v) Fetal Bovine Serum (Hyclone), 100 U/ml penicillin and 100 U/ml streptomycin (Gibco). Cells were passaged by trypsinizing with 0.25% Trypsin-EDTA (Invitrogen) and plated at a ratio of 1:6. To induce neuronal differentiation, 10 μM all-trans retinoic acid (Sigma) was added to media once cells reached a density of ~50%. During the 8-day differentiation time course media was changed every 2–3 days.

### Western blotting analysis

For total lystate analysis, cells were resuspended in RIPA buffer (25 mM Tris·HCl pH 7.6, 150 mM NaCl, 1% NP40, 1% sodium deoxycholate, 0.1% SDS, 2 μg/mL Aprotinin, 1 μg/mL Leupeptin, 10 mM PMSF). Lysates were incubated for 15 min on ice and cell membranes disrupted mechanically by syringing 5 times with 23G narrow gauge needle and sonicating 3 × 2 s at high power. Lysates were incubated on ice for another 15 min and lysates pre-cleared by centrifugation at 14,000 rpm 4 °C 30 min to remove cellular debris. For analysis of nuclear fraction, lysates were resuspended in an equal volume of Buffer C (20 mM HEPES pH 7.9, 0.2 mM EDTA, 1.5 mM MgCl2, 20% glycerol, 420 mM NaCl, 2 μg/mL Aprotinin, 1 μg/mL Leupeptin, 10 mM PMSF) and dounced 20 times with tight pestle type B (Tight). Lysates were incubated for 45 min rotating to dissociated chromatin-bound proteins and pre-cleared by centrifugation at 14,000 rpm 4C for 30 min to remove cellular debris and intact chromatin. Immunoblotting was performed using antibodies described in Supplementary Table S3.

### Immunoprecipitation

10 μg antibody was coupled to 50 μL packed Protein A beads (Sigma P9424) by incubation in 1 mL PBS (0.1% Tween-20) at 4 °C rotating overnight. Beads were collected by centrifugation at 1700 × g for 3 min and washed twice in 1 mL 0.2 M Sodium Borate pH 9.0. Antibodies were then crosslinked to beads by incubation in 1 mL 0.2 M Sodium Borate pH 9.0 (20 mM dimethyl pimelimidate dihydrochloride) at room temperature rotating for 30 min. Reaction was terminated by washing beads once in 1 mL 0.2 M Ethanolamine pH 8.0 and incubating for 2 hr at room temperature rotating in 1 mL 0.2 M Ethanolamine pH 8.0. Beads were washed twice in Buffer C100 and blocked for 1 h min 4 °C rotating in Buffer C100 (0.1 mg/mL Insulin (Sigma, I9278), 0.2 mg/mL Chicken egg albumin (Sigma A5503), 0.1% (v/v) fish skin gelatin (Sigma G7041). Antibody-crosslinked beads were incubated with nuclear lysates, in the presence of 250 U/mL Benzonase nuclease, at 4 °C rotating overnight and washed 5 × 5 min in Buffer C100 (0.02% NP-40). After the final wash, beads were resuspended in 50 μL 2x SDS sample buffer. Immunoprecipitated material was eluted by boiling for 5 min with shaking and associated proteins were separated by SDS-PAGE and analysed by immunoblotting.

### Ectopic Immunoprecipitations FLAG-TAG

Immunoprecipitations (IPs) were performed on nuclear protein lysates prepared in low salt buffer containing protease inhibitors (150 mM NaCl, 50 mM TRIS pH 8.0, 1 mM EDTA, 1% NP40, 1 μg/ml aprotinin, 10 μg/ml leupeptin, 1 mM PMSF). IPs of FLAG tagged proteins were performed using M2 anti- FLAG agarose and mouse IgG agarose (Sigma) overnight at 4 °C. Elution of FLAG tagged proteins was performed at 4 °C using 250 ug/ml of 3xFLAG peptide (Sigma) in 0.05% NP40 with horizontal shaking. Eluted protein fractions were separated by SDSPAGE and analyzed by western blot or liquid chromatography mass spectrometry.

### Gel filtration column chromatography

The Superose^TM^ 6 10/300 GL gel filtration column (GE Healthcare) was equilibrated with one column volume of running buffer (20 mM Tris pH 8.0, 10% Glycerol, 175 mM NaCl, 0.5 mM DTT, 1 mM PMSF). 300–500 μg of total nuclear protein (prepared as described above) was injected and run through column at 0.35 mL/min. 1 mL fractions were collected and protein was concentrated by incubation with 4 μL StrataClean resin (Agilent Technologies) for 1 hour at room temperature. Resin was collected by centrifugation at 5000 rpm for 3 min and protein was eluted by boiling in 20 μL 2X SDS sample buffer for 5 min shaking at 1400 rpm. Eluted protein analysed by SDS-PAGE and immunoblotting.

### In-solution trypsin digest and mass spectrometry

Proteins were treated with trypsin as described^46^. Peptide samples were introduced Q Exactive mass spectrometer via an EASY-nLC 1000 UHPLC system (Thermo Fisher) coupled to an in-house packed C18 column (New Objective). Parent ion spectra (MS1) were measured at resolution 70,000, AGC target 3e6. Tandem mass spectra (MS2; up to 10 scans per duty cycle) were obtained at resolution 17,500, AGC target 5e4, collision energy of 25. Data were processed using MaxQuant version 1.3.0.5^47^ using the human UniProt database (release 2013_12; 67,911 entries). The following search parameters were used: Fixed Mod: carbamidomethylation; Variable Mods: methionine oxidation; Trypsin/P digest enzyme; Precursor mass tolerances 6 ppm; Fragment ion mass tolerances 20 ppm; Peptide FDR 1%; Protein FDR 1%.

### Data analysis

The data network design of protein-protein interactions (interactome network) between PCGF1, and the potential interactors in NT2 cells was performed using Cytoscape software, version 3.1.1. For this purpose, we used the interactome data of *Homo sapiens*. The interactome networks obtained from this first screening were analyzed with BiNGO, a Cytoscape plugin to assess overrepresentation of gene ontology categories in biological networks^22^. Volcano plots of LC-MS/MS data of PCGF1, BCOR, DPPA4, NANOG and OCT4 immunoprecipitations in NT2 cells were performed using Perseus software, version 1.4^20^.

### Real-time quantitative PCR

Extracted RNA was used to generate cDNA by reverse transcriptase PCR using the TaqMan Reverse Transcription kit (Applied Biosytems). Relative mRNA expression levels were determined using the SYBR Green I detection chemistry on LightCycler 480II Real–Time PCR System (Roche). The ribosomal constituent RPO was used as normalizing gene. Primers are listed in Supplementary Table S3.

### Lentivirus production and shRNA treatement

Lentiviral particles were produced and used in the transduction in cells target as previously described^48^,^49^. shRNA pLKO.1 vectors expressing control (Scrambled) or *PCGF1*-specific shRNA sequences were purchased from Sigma (Supplementary Table S4).

## Additional Information

**How to cite this article**: Oliviero, G. *et al.* The variant Polycomb Repressor Complex 1 component PCGF1 interacts with a pluripotency sub-network that includes DPPA4, a regulator of embryogenesis. *Sci. Rep.* **5**, 18388; doi: 10.1038/srep18388 (2015).

## Supplementary Material

Supplementary Information

## References

[b1] Chen T. & Dent S. Y. Chromatin modifiers and remodellers: regulators of cellular differentiation. Nature reviews. Genetics 15, 93–106, 10.1038/nrg3607 (2014). PMC3999985 24366184

[b2] Simon J. A. & Kingston R. E. Occupying chromatin: Polycomb mechanisms for getting to genomic targets, stopping transcriptional traffic, and staying put. Molecular cell 49, 808–824, 10.1016/j.molcel.2013.02.013 (2013). 23473600 PMC3628831

[b3] Simon J. A. & Kingston R. E. Mechanisms of polycomb gene silencing: knowns and unknowns. Nature reviews. Molecular cell biology 10, 697–708, 10.1038/nrm2763 (2009). 19738629

[b4] Schuettengruber B., Chourrout D., Vervoort M., Leblanc B. & Cavalli G. Genome regulation by polycomb and trithorax proteins. Cell 128, 735–745, 10.1016/j.cell.2007.02.009 (2007). 17320510

[b5] Schuettengruber B. & Cavalli G. Recruitment of polycomb group complexes and their role in the dynamic regulation of cell fate choice. Development 136, 3531–3542, 10.1242/dev.033902 (2009). 19820181

[b6] Wang H. *et al.* Role of histone H2A ubiquitination in Polycomb silencing. Nature 431, 873–878, 10.1038/nature02985 (2004). 15386022

[b7] Cao R., Tsukada Y. & Zhang Y. Role of Bmi-1 and Ring1A in H2A ubiquitylation and Hox gene silencing. Molecular cell 20, 845–854, 10.1016/j.molcel.2005.12.002 (2005). 16359901

[b8] Grau D. J., Antao J. M. & Kingston R. E. Functional dissection of Polycomb repressive complex 1 reveals the importance of a charged domain. Cold Spring Harbor symposia on quantitative biology 75, 61–70, 10.1101/sqb.2010.75.056 (2010). 21502414

[b9] Saurin A. J., Shao Z., Erdjument-Bromage H., Tempst P. & Kingston R. E. A Drosophila Polycomb group complex includes Zeste and dTAFII proteins. Nature 412, 655–660, 10.1038/35088096 (2001). 11493925

[b10] Gao Z. *et al.* PCGF homologs, CBX proteins, and RYBP define functionally distinct PRC1 family complexes. Molecular cell 45, 344–356, 10.1016/j.molcel.2012.01.002 (2012). 22325352 PMC3293217

[b11] Shao Z. *et al.* Stabilization of chromatin structure by PRC1, a Polycomb complex. Cell 98, 37–46, 10.1016/S0092-8674(00)80604-2 (1999). 10412979

[b12] Endoh M. *et al.* Histone H2A mono-ubiquitination is a crucial step to mediate PRC1-dependent repression of developmental genes to maintain ES cell identity. PLoS genetics 8, e1002774, 10.1371/journal.pgen.1002774 (2012). 22844243 PMC3405999

[b13] Levine S. S. *et al.* The core of the polycomb repressive complex is compositionally and functionally conserved in flies and humans. Molecular and cellular biology 22, 6070–6078 (2002). 12167701 10.1128/MCB.22.17.6070-6078.2002 PMC134016

[b14] Gearhart M. D., Corcoran C. M., Wamstad J. A. & Bardwell V. J. Polycomb group and SCF ubiquitin ligases are found in a novel BCOR complex that is recruited to BCL6 targets. Molecular and cellular biology 26, 6880–6889, 10.1128/MCB.00630-06 (2006). 16943429 PMC1592854

[b15] Farcas A. M. *et al.* KDM2B links the Polycomb Repressive Complex 1 (PRC1) to recognition of CpG islands. eLife 1, e00205, 10.7554/eLife.00205 (2012). 23256043 PMC3524939

[b16] Long H. K., Blackledge N. P. & Klose R. J. ZF-CxxC domain-containing proteins, CpG islands and the chromatin connection. Biochemical Society transactions 41, 727–740, 10.1042/BST20130028 (2013). 23697932 PMC3685328

[b17] Lee V. M. & Andrews P. W. Differentiation of NTERA-2 clonal human embryonal carcinoma cells into neurons involves the induction of all three neurofilament proteins. The Journal of neuroscience: the official journal of the Society for Neuroscience 6, 514–521 (1986). 2419526 10.1523/JNEUROSCI.06-02-00514.1986 PMC6568536

[b18] Wu X., Johansen J. V. & Helin K. Fbxl10/Kdm2b recruits polycomb repressive complex 1 to CpG islands and regulates H2A ubiquitylation. Molecular cell 49, 1134–1146, 10.1016/j.molcel.2013.01.016 (2013). 23395003

[b19] Blackledge N. P. *et al.* Variant PRC1 complex-dependent H2A ubiquitylation drives PRC2 recruitment and polycomb domain formation. Cell 157, 1445–1459, 10.1016/j.cell.2014.05.004 (2014). 24856970 PMC4048464

[b20] Bracken A. P., Dietrich N., Pasini D., Hansen K. H. & Helin K. Genome-wide mapping of Polycomb target genes unravels their roles in cell fate transitions. Genes & development 20, 1123–1136, 10.1101/gad.381706 (2006). 16618801 PMC1472472

[b21] Hatzi K. *et al.* A hybrid mechanism of action for BCL6 in B cells defined by formation of functionally distinct complexes at enhancers and promoters. Cell Reports 4, 578–88. 10.1016/j.celrep.2013.06.016. (2013). 23911289 PMC3854650

[b22] Maere S., Heymans K. & Kuiper M. BiNGO: a Cytoscape plugin to assess overrepresentation of gene ontology categories in biological networks. Bioinformatics 21, 3448–3449, 10.1093/bioinformatics/bti551 (2005). 15972284

[b23] Francis N. J., Saurin A. J., Shao Z. & Kingston R. E. Reconstitution of a functional core polycomb repressive complex. Molecular cell 8, 545–556 (2001). 11583617 10.1016/s1097-2765(01)00316-1

[b24] Ow J. R. *et al.* Patz1 regulates embryonic stem cell identity. Stem cells and development 23, 1062–1073, 10.1089/scd.2013.0430 (2014). 24380431

[b25] Maldonado-Saldivia J. *et al.* Dppa2 and Dppa4 are closely linked SAP motif genes restricted to pluripotent cells and the germ line. Stem cells 25, 19–28, 10.1634/stemcells.2006-0269 (2007). 16990585

[b26] Kashyap V. *et al.* Regulation of stem cell pluripotency and differentiation involves a mutual regulatory circuit of the NANOG, OCT4, and SOX2 pluripotency transcription factors with polycomb repressive complexes and stem cell microRNAs. Stem cells and development 18, 1093–1108, 10.1089/scd.2009.0113 (2009). 19480567 PMC3135180

[b27] Li H., Fan R., Sun M., Jiang T. & Gong Y. Nspc1 regulates the key pluripotent Oct4-Nanog-Sox2 axis in P19 embryonal carcinoma cells via directly activating Oct4. Biochemical and biophysical research communications 440, 527–532, 10.1016/j.bbrc.2013.09.095 (2013). 24113379

[b28] van Nuland R. *et al.* Quantitative dissection and stoichiometry determination of the human SET1/MLL histone methyltransferase complexes. Molecular and cellular biology 33, 2067–2077, 10.1128/MCB.01742-12 (2013). 23508102 PMC3647974

[b29] He J. *et al.* Kdm2b maintains murine embryonic stem cell status by recruiting PRC1 complex to CpG islands of developmental genes. Nature Cell Biology 15, 373–84, 10.1038/ncb2702 (2013). PMC4078788 23502314

[b30] Boyer L. A. *et al.* Polycomb complexes repress developmental regulators in murine embryonic stem cells. Nature 441, 349–353, 10.1038/nature04733 (2006). 16625203

[b31] Vidal M. Role of polycomb proteins Ring1A and Ring1B in the epigenetic regulation of gene expression. The International journal of developmental biology 53, 355–370, 10.1387/ijdb.082690mv (2009). 19412891

[b32] Bortvin A. *et al.* Incomplete reactivation of Oct4-related genes in mouse embryos cloned from somatic nuclei. Development 130, 1673–1680 (2003). 12620990 10.1242/dev.00366

[b33] Masaki H., Nishida T., Kitajima S., Asahina K. & Teraoka H. Developmental pluripotency-associated 4 (DPPA4) localized in active chromatin inhibits mouse embryonic stem cell differentiation into a primitive ectoderm lineage. The Journal of biological chemistry 282, 33034–33042, 10.1074/jbc.M703245200 (2007). 17855347

[b34] Madan B. *et al.* The pluripotency-associated gene Dppa4 is dispensable for embryonic stem cell identity and germ cell development but essential for embryogenesis. Molecular and cellular biology 29, 3186–3203, 10.1128/MCB.01970-08 (2009). 19332562 PMC2682008

[b35] Tung P. Y., Varlakhanova N. V. & Knoepfler P. S. Identification of DPPA4 and DPPA2 as a novel family of pluripotency-related oncogenes. Stem cells 31, 2330–2342, 10.1002/stem.1526 (2013). 23963736 PMC3922068

[b36] Rolland T. *et al.* A proteome-scale map of the human interactome network. Cell 159, 1212–1226, 10.1016/j.cell.2014.10.050 (2014). 25416956 PMC4266588

[b37] Qin J. *et al.* The polycomb group protein L3mbtl2 assembles an atypical PRC1-family complex that is essential in pluripotent stem cells and early development. Cell stem cell 11, 319–332, 10.1016/j.stem.2012.06.002 (2012). 22770845 PMC3647456

[b38] Trojer P. *et al.* L3MBTL2 protein acts in concert with PcG protein-mediated monoubiquitination of H2A to establish a repressive chromatin structure. Molecular cell 42, 438–450, 10.1016/j.molcel.2011.04.004 (2011). 21596310 PMC3142354

[b39] Fukuda T., Tokunaga A., Sakamoto R. & Yoshida N. Fbxl10/Kdm2b deficiency accelerates neural progenitor cell death and leads to exencephaly. Molecular and cellular neurosciences 46, 614–624, 10.1016/j.mcn.2011.01.001 (2011). 21220025

[b40] He J., Kallin E. M., Tsukada Y. & Zhang Y. The H3K36 demethylase Jhdm1b/Kdm2b regulates cell proliferation and senescence through p15(Ink4b). Nature structural & molecular biology 15, 1169–1175, 10.1038/nsmb.1499 (2008). PMC2612995 18836456

[b41] Tzatsos A., Pfau R., Kampranis S. C. & Tsichlis P. N. Ndy1/KDM2B immortalizes mouse embryonic fibroblasts by repressing the Ink4a/Arf locus. Proceedings of the National Academy of Sciences of the United States of America 106, 2641–2646, 10.1073/pnas.0813139106 (2009). 19202064 PMC2650317

[b42] Knoepfler P. S. Deconstructing stem cell tumorigenicity: a roadmap to safe regenerative medicine. Stem cells 27, 1050–1056, 10.1002/stem.37 (2009). 19415771 PMC2733374

[b43] Lobo N. A., Shimono Y., Qian D. & Clarke M. F. The biology of cancer stem cells. Annual review of cell and developmental biology 23, 675–699, 10.1146/annurev.cellbio.22.010305.104154 (2007). 17645413

[b44] Nguyen L. K. *et al.* Switches, excitable responses and oscillations in the Ring1B/Bmi1 ubiquitination system. PLoS computational biology 7, e1002317, 10.1371/journal.pcbi.1002317 (2011). 22194680 PMC3240587

[b45] Guo W. J., Datta S., Band V. & Dimri G. P. Mel-18, a polycomb group protein, regulates cell proliferation and senescence via transcriptional repression of Bmi-1 and c-Myc oncoproteins. Molecular biology of the cell 18, 536–546, 10.1091/mbc.E06-05-0447 (2007). 17151361 PMC1783768

[b46] Wisniewski J. R., Zougman A., Nagaraj N. & Mann M. Universal sample preparation method for proteome analysis. Nature methods 6, 359–362, 10.1038/nmeth.1322 (2009). 19377485

[b47] Cox J. & Mann M. MaxQuant enables high peptide identification rates, individualized p.p.b.-range mass accuracies and proteome-wide protein quantification. Nature biotechnology 26, 1367–1372, 10.1038/nbt.1511 (2008). 19029910

[b48] Brien G. L. *et al.* Polycomb PHF19 binds H3K36me3 and recruits PRC2 and demethylase NO66 to embryonic stem cell genes during differentiation. Nature structural & molecular biology 19, 1273–1281, 10.1038/nsmb.2449 (2012). 23160351

[b49] Pasini D. *et al.* Coordinated regulation of transcriptional repression by the RBP2 H3K4 demethylase and Polycomb-Repressive Complex 2. Genes & development 22, 1345–1355, 10.1101/gad.470008 (2008). 18483221 PMC2377189

